# Tracking phases of the fall armyworm (*Spodoptera frugiperda*) invasion across multiple continents using news media

**DOI:** 10.1007/s10530-026-03887-3

**Published:** 2026-07-20

**Authors:** Kathryn Bjorklund, Melissa A. Barton, Stefan Daume, Peter Søgaard Jørgensen

**Affiliations:** 1 Stockholm Resilience Centre, Stockholm University, Stockholm, Sweden; 2 Beijer Institute of Ecological Economics, The Royal Swedish Academy of Sciences, Stockholm, Sweden; 3 Global Economic Dynamics and the Biosphere, The Royal Swedish Academy of Sciences, Stockholm, Sweden

**Keywords:** Social-ecological impacts, Fall armyworm, *Spodoptera frugiperda*, Text analysis, Digital news articles

## Abstract

Understanding cascading social-ecological impacts of emerging pests is key for addressing the challenges they introduce in an ever more interconnected and rapidly changing world. Here we examine shifts in news media discourse surrounding the fall armyworm invasion across Africa, Asia, and Oceania to investigate such cascades. We apply topic modeling to 570 news articles to quantify spatial and temporal changes in the prevalence of 33 topics related to fall armyworm invasion discourse. We grouped these topics into broader thematic categories and determined their prevalence during three general invasion phases. We found that the sequence of prevalent thematic categories was consistent across news articles from the three continents analyzed. The first years of invasion discourse are dominated by topics related to a preparation-oriented ‘Surveillance’ phase, followed by those related to an ‘Impact’ phase, and finally a response-centered ‘Control’ phase. However, the duration of the ‘Impact’ phase differs by continent, lasting 3 of the 7 years analyzed in Africa, but only 1 of 5 and 1 of 3 in Asia and Oceania, respectively. Also in Oceania’s news discourse, invasion preparation features more prominently with a relative theme proportion of 0.722 in the first year after detection (Africa = 0.387, Asia = 0.259). These differences in phase duration and emphasis align with described patterns of the invasion progression and outcomes across regions. These regional variations highlight the potential of text analysis of news media for tracking cascading dynamics of emerging pests. In turn, these approaches can inform more targeted management and mitigation strategies.

**Supplementary Information:**

The online version contains supplementary material available at https://doi.org/10.1007/s10530-026-03887-3.

## Introduction

Emergent pest species can have far-reaching consequences on society that extend beyond their immediate impacts. The coffee leaf rust (*Hemileia vastatrix*) epidemic in Central America is a recent example of this with the devastation of coffee crops prompting a mass domestic and international migration of smallholder farmers (Dupre et al. 2022). As emerging pest species generate cascading effects across ecological and social domains, there remains a need for methods that can track not only biological spread, but also how impacts and responses unfold over time.

Digital news media analysis offers one way to study these unfolding dynamics. Media coverage chronicles events as they occur across regions and captures the public sentiment surrounding them, offering a valuable resource for understanding the societal dimensions of biological invasions. It can provide scientists and conservation practitioners with timely insights into where problem species are being detected, what impacts they are producing, who they are affecting, and how groups are responding, revealing information gaps that can support the tailoring of prevention and management strategies (Tateosian et al. 2023). Web-based news analysis has already been successfully applied in several instances to develop event-based surveillance systems and early detection tools for plant and animal diseases. Examples include the Global Public Health Intelligence Network (GPHIN), developed by Health Canada for the World Health Organization (WHO) in 1997, which monitors diverse digital, multilingual global media sources and notably played a role in the early detection of the SARS outbreak of 2002 (Galaz et al. 2010; Bloodworth et al. 2021), as well the Medical Intelligence System (MedISys) (Authority (EFSA) 2022), HealthMap (Freifeld et al. 2008), and the Platform for Automated extraction of Disease Information from the web (PADI-web) (Roche et al. 2024). These tools illustrate how web-based information can complement official reporting to detect early warning signals and facilitate timely intervention. However, there remains scope to extend such approaches past detecting the presence of pests or diseases, toward tracking their downstream social-ecological impacts, including who is affected, how impacts are discussed, and which institutions respond.

**Fig. 1:**
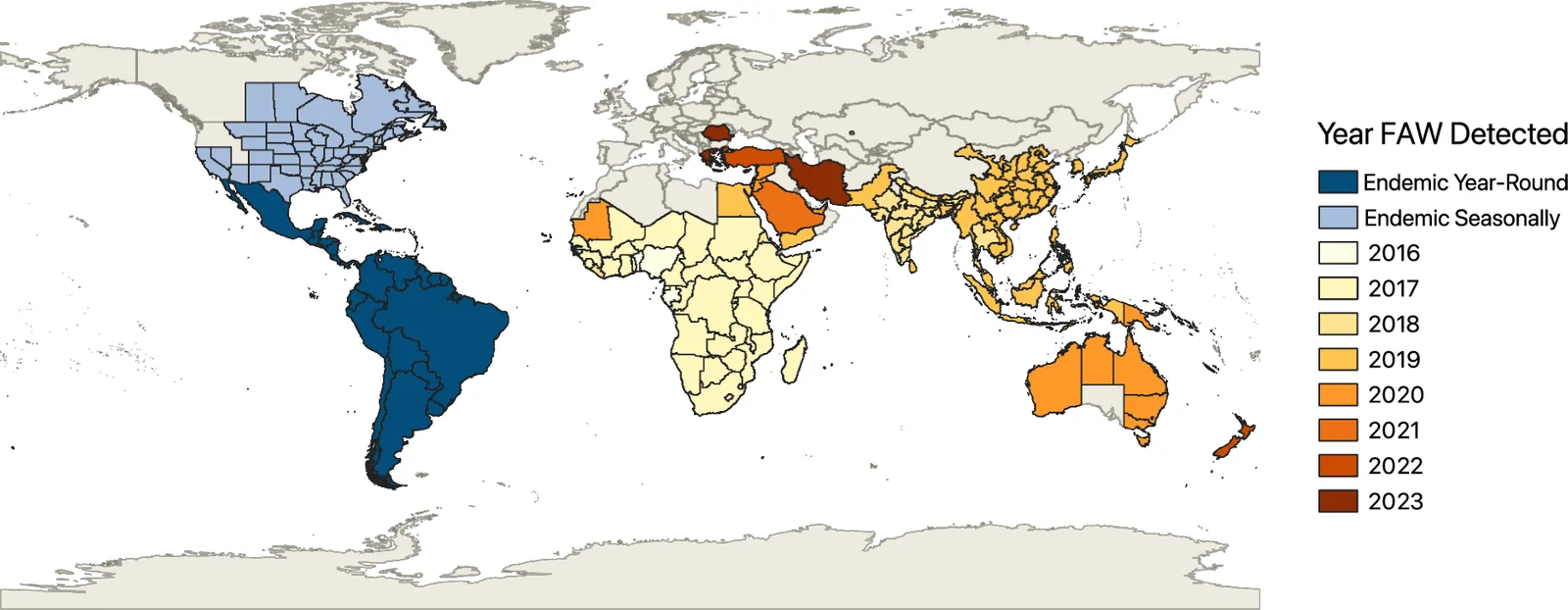
Map of spread of fall armyworm (FAW). The boundaries of larger countries India, China and Australia have been subdivided to depict more specific areas targeted. FAW data compiled from various sources can be found in Appendix 1 in supplementary material. Map was constructed using QGIS version 3.36.1 with spatial data from DIVA-GIS (https://diva-gis.org/) and Natural Earth (https://www.naturalearthdata.com/)

We examine this potential through the case of the fall armyworm (*Spodoptera frugiperda*; FAW), an agricultural pest native to the Americas that was introduced to Africa in 2016 (Lu and Adang 1996), likely as a stowaway on commercial aircraft (Day et al. 2017). In part due to its ability to migrate long distances on prevailing winds (Day et al. 2017), FAW spread rapidly across three continents over the span of 6 years (Fig. 1), contributing to widespread crop damage, primarily affecting maize (Prasanna et al. 2022). FAW in Africa is responsible for an estimated 9.4 billion USD in yield losses per annum, making it the most damaging invasive species on the continent (Eschen et al. 2021). These losses threaten the food security and livelihoods of smallholder farmers in particular (Devi 2018; Tambo et al. 2020). The rapid spread of FAW, its regional progression, and its documented ecological and social consequences make it a useful case for examining how invasion-related impacts and responses are represented in news media over time.

In this paper, we use the FAW invasion to develop and demonstrate a mixed-methods approach for analyzing digital news media discourse around emerging pest species as a means to study representations of impacts. Building on previous uses of web-based media for surveillance, we apply structural topic modelling to examine how themes in FAW news coverage vary across regions and over the course of the invasion. Specifically, we aim to: (1) identify the themes that emerge in public discourse about the fall armyworm and examine how they evolve over time (2) assess how different regions vary in the emphasis they place on these themes throughout the invasion timeline, and (3) evaluate how the temporal patterns in public discourse correspond to phases of biological invasion described in academic literature.

## Methods

### Data collection

We selected digital news media as the data source for our investigation into the fall armyworm invasion, leveraging its real-time nature and broad coverage. News media sources also provide diverse perspectives on the issue and serve as gauges of public interest and concern (Tateosian et al. 2023).

We extracted news articles from the freely accessible Google News platform, which aggregates news articles from thousands of sources worldwide and stores web news content extending back to 2003. We retrieved URLs of articles published between 1 January 2016 and 9 December 2022 from the Google News RSS feed. We used the Latin name “*Spodoptera frugiperda*” and the English common name “armyworm” as search terms to obtain approximately 1,100 URLs for English-language articles. We used the tool Extractor API (https://extractorapi.com/) to obtain the full texts, domains, and publication dates.

### Structural topic model

We used a structural topic model (STM), a probabilistic topic modelling approach that facilitates the identification of latent topics within large text collections. Topic modelling identifies topics from patterns of word co-occurrence in a corpus, estimating both the prevalence of topics within documents and the probability with which words are associated with each topic. Topic discovery is unsupervised, meaning the model infers topics directly without predefined categories. STM uses a Bayesian framework to estimate how topic prevalence varies with document-level metadata as covariates, enabling deeper contextual analysis (Roberts et al. 2019). RStudio (R version 4.4.0) was used to implement the model.

To support this approach, we annotated a news article corpus with metadata. First, we manually reviewed each article to identify its focal country or region. When the main share of described events, impacts, or responses centered on a single location, typically linked to two-thirds or more of the article’s narrative, we assigned that country or region as the focal location. Articles that distributed attention across multiple places without a clear dominant location were coded at a broader regional level. Each focal location was then linked to its corresponding continent to enable geographic analysis of topics and themes. Second, we established a FAW entry date for each country referenced, drawing on multiple sources (Appendix 1 in supplementary material), and calculated an “invasion year” (the number of years at time of publication since FAW first entered a country) for each article by subtracting the country-specific FAW entry date from the article publication date. We used this invasion year as metadata to track the evolution of the themes in public discourse as the FAW invasion progressed. Finally, we eliminated duplicate articles and those regarding countries where FAW is considered endemic. The refined news article corpus encompasses content from 45 of the 87 countries where FAW has emerged. Although FAW reached several West Asian countries later than other parts of Asia, the number of news articles from this subregion was relatively small. We therefore grouped West Asia with the broader Asian region for analysis.

Using the **stm** package, we fitted the STM using complete news articles (N = 570) as documents (Roberts et al. 2019). We included continent and invasion year as covariates in order to explore regional variances and thematic shifts over time in FAW discourse. We used the **quanteda** package for text pre-processing (Benoit et al. 2018). We removed English stop words, a custom list of geographic terms (country, city, and ISO code names from the SimpleMaps World Cities Database), and FAW-specific terms to refine topic analysis by eliminating ubiquitous, and thus, less informative words. We selected a K value of 33 topics for the STM based on iterative model diagnostics and interpretation across models ranging from 10 to 100 topics (Appendix 2 in supplementary material). We prioritized semantic coherence and granularity to produce topics that were meaningfully labelable and distinct enough to capture different types of reported activities and responses.

### Interpreting the structural topic model

In STM, like other topic models, topics are characterized by the probability (Prob) of words belonging to a specific topic, indicating the strength of association. In addition, STM offers a metric quantifying the combined frequency and exclusivity of words within a topic (FREX) (Roberts et al. 2019). We interpreted each topic generated by the STM by examining its Prob and FREX terms, supplemented by manual review of the five most representative documents for each topic (Appendix 3 in supplementary material). We then categorized topics into nine broader themes: (1) ‘Invasive Species’ to capture more general and introductory discussion of FAW, (2) ‘Surveillance and Prevention’ to group discourse surrounding proactive measures of different regions, (3) ‘Research and Development’ to group evidence of investment in solution-oriented efforts, (4) ‘Conventional Control’ and (5) ‘Agroecological Control’ to distinguish between reactive approaches to FAW management across regions, (6) ‘Government’ to evaluate involvement of governmental bodies in addressing FAW, (7) ‘Agriculture’ to illustrate wide-ranging, sectoral discourse around FAW, and (8) ‘Crop Impacts’ and (9) ‘Social Impacts’ to differentiate the types of impacts observed in different regions (Table 1). Additional details on the theme creation process, topic descriptions, dominant topic assignment across articles, and covariate-topic relationships are provided in Appendices 4–7 of supplementary material.

**Table 1:** Theme names, decriptions, and associated topics

Theme	Theme description	Topic
Agriculture	Impacts of FAW on crop production, farming systems, and agricultural development	(5) Domestic Agriculture
		(15) Agricultural Sector
Government	Role of local, national, and international institutions in managing the impacts of FAW	(8) Agricultural Empowerment Programs
		(9) Financial Aid
		(24) Local Government Involvement
		(27) Agriculture Department Work
		(30) National Government Budgeting and Programming
Invasive species	Shifts in temperature, precipitation, and other environmental factors in relation to phenology, range and population of invasive pests	(17) Climate and Invasive Pests
		(26) Invasive Pest Species
Social impacts	Impacts of FAW on agricultural workers and communities at large	(2) Socioeconomic Impacts
		(7) Food Insecurity
		(20) Farmer Burdens
Crop impacts	Impacts of FAW on physical plants and crop counts	(4) Crop Losses
		(11) Damage
		(18) Productivity and Yields
Agroecological control	Diversity, effectiveness, and broader implications of agroecological pest management techniques	(13) Homemade Pest Control Remedies
		14) Biological Pest Control Research
		(19) Natural Enemies
		(25) Integrated Pest Management
		(32) Agroecological Pest Control Methods
Research & development	Exploration in the ways in which technological innovations are transforming the field of pest management	(3) Invasive Pest Species Research
		(23) Technology
		(29) Research and Development/Innovation
Conventional control	Diversity, effectiveness, and broader implications of conventional pest management techniques	(10) Pesticide Research
		(12) Biotechnology/Gene Editing FAW
		(21) Pesticide Procurement
		(22) Transgenic Crops/Genetically Modified Organisms
		(31) Seed Treatments
Surveillance & Prevention	Strategies and tools used to prevent the spread of FAW and detect its presence in agricultural settings	(1) Spread of the fall armyworm (FAW)
		(6) Drought/Weather Patterns and FAW
		(16) Biosecurity
		(28) Insect Monitoring
		(33) Invasion Preparation

### Prediction theme prevalence across biological invasion phases

**Fig. 2:**
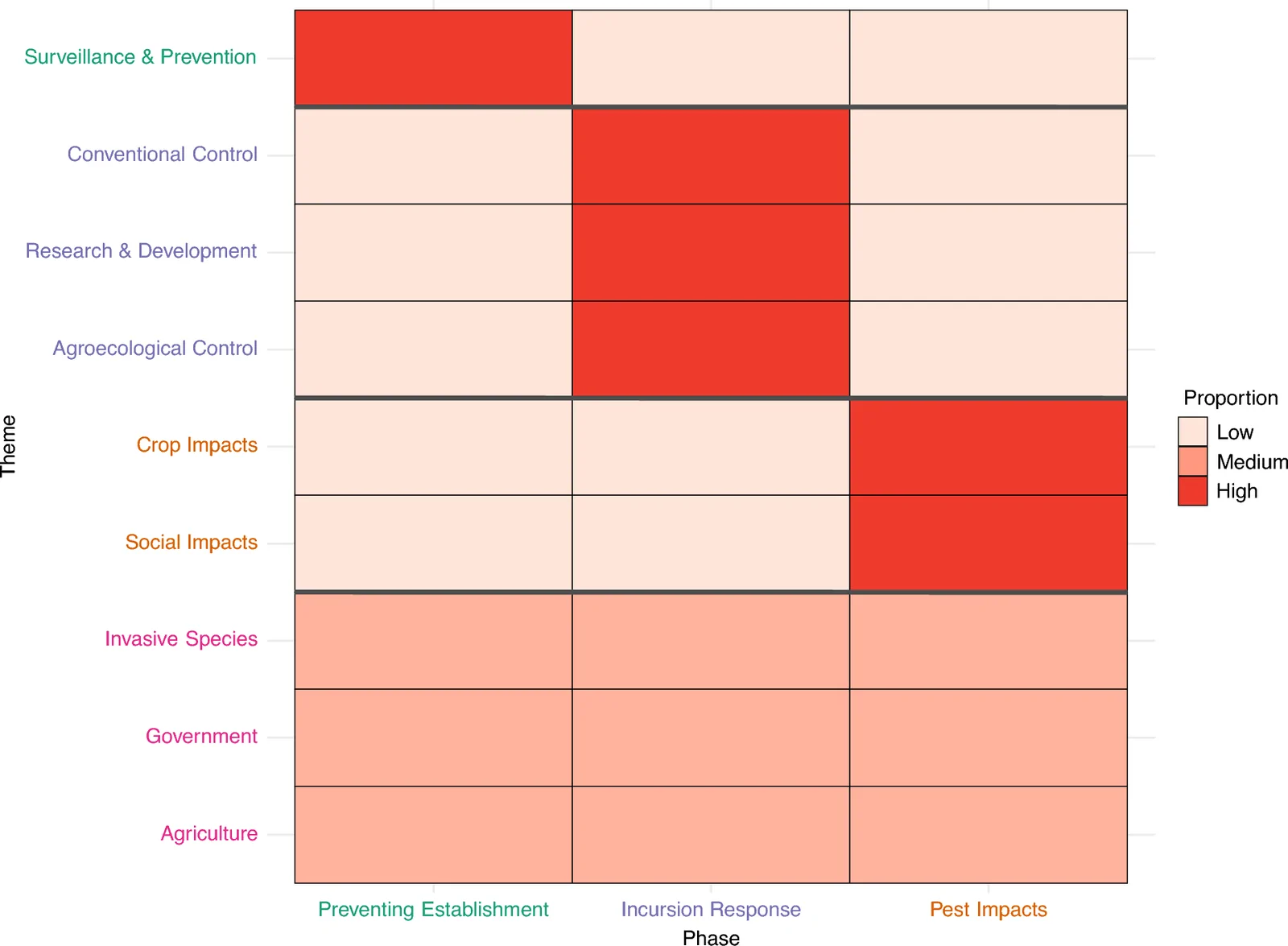
Overview of predicted theme distributions mapped onto the phases of the biological invasion process described by Welsh et al. (2021). Themes such as ‘Surveillance and Prevention’ corresponds strongly with the initial ‘Preventing Establishment’ phase; ‘Conventional Control,’ ‘Research and Development,’ and ‘Agroecological Control’ with the ‘Incursion Response’ phase; ‘Crop Impacts’ and ‘Social Impacts’ apply to the ‘Pest Impacts’ phase; and ‘Invasive Species,’ ‘Government,’ and ‘Agriculture’ constitute cross-cutting themes that span multiple stages of the invasion process

We further contextualized identified themes by formulating predictions about their likely occurrence and distribution across the phases of the biological invasion process, as described by Welsh et al. (2021) (Fig. 2). In the first phase ‘Preventing Establishment’, we expect the theme ‘Surveillance and Prevention’ to be prevalent as it focuses on proactive measures used to prevent and monitor potential invasions.

In the second phase, following the detection of a pest, an ‘Incursion Response’ occurs, during which efforts to identify, eradicate, contain, and manage the pest invasion take place, as presented by Welsh et al. (2021). We expect the themes ‘Conventional Control,’ ‘Agroecological Control,’ and ‘Research and Development’ to be prevalent in this phase, as many regions initially rely on synthetic insecticides to address immediate threats of pests (Bale et al. 2008; Brévault and Bouyer 2014) or employ sustainable pest management trials and practices to reduce potential damage. Similarly, increased funding for research and development and scientific advancements are likely to occur in response to the invasion (Fig. 2).

Lastly, Welsh et al.’s biological invasion process includes a ‘Pest Impacts’ phase, where a pest’s direct and indirect effects, such as yield losses, economic burdens, and social consequences like job losses and market disruptions, are experienced (Welsh et al. 2021). We categorized the ‘Crop Impacts’ and ‘Social Impacts’ themes as relevant to this phase, as they address the broader consequences of the invasion (Fig. 2).

Finally, we expect the themes ‘Invasive Species,’ ‘Government,’ and ‘Agriculture’ to occur across the three phases (Fig. 2), as they encompass transitional or overarching elements such as pest biology, governmental involvement, and agricultural metrics.

### Calculating the prevalence of themes by continent and by continent over time

**Fig. 3:**
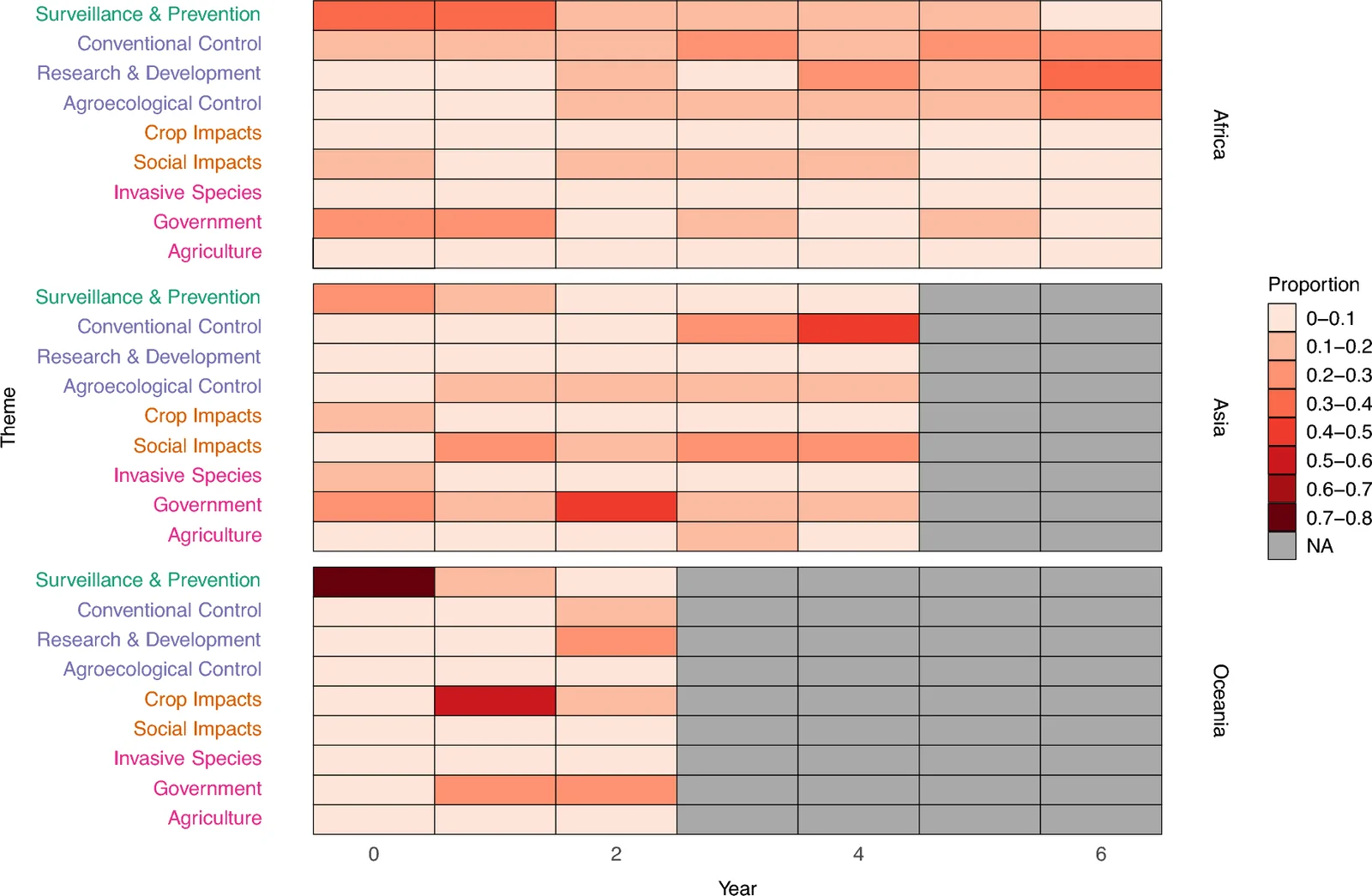
Thematic prevalence for articles involving Africa (N = 300), Asia (N = 214) and Oceania (N = 55) over year since fall armyworm entry

To capture contextual variability and reduce noise in the model, we aggregated news articles by ‘continent’ and by both ‘continent’ and ‘invasion year.’ For each grouping, we calculated the mean topic proportions to understand how dominant or frequent certain topics were in different continents or during different years. We then added the nine predefined themes as a variable to the groupings, which allowed for the categorization of related topics into broader themes. We then summed the mean topic proportions for topics that fell under the same theme to calculate the cumulative proportions of each theme (Fig. 3).

### Calculating biological invasion phase of best fit by continent over time

**Fig. 4:**
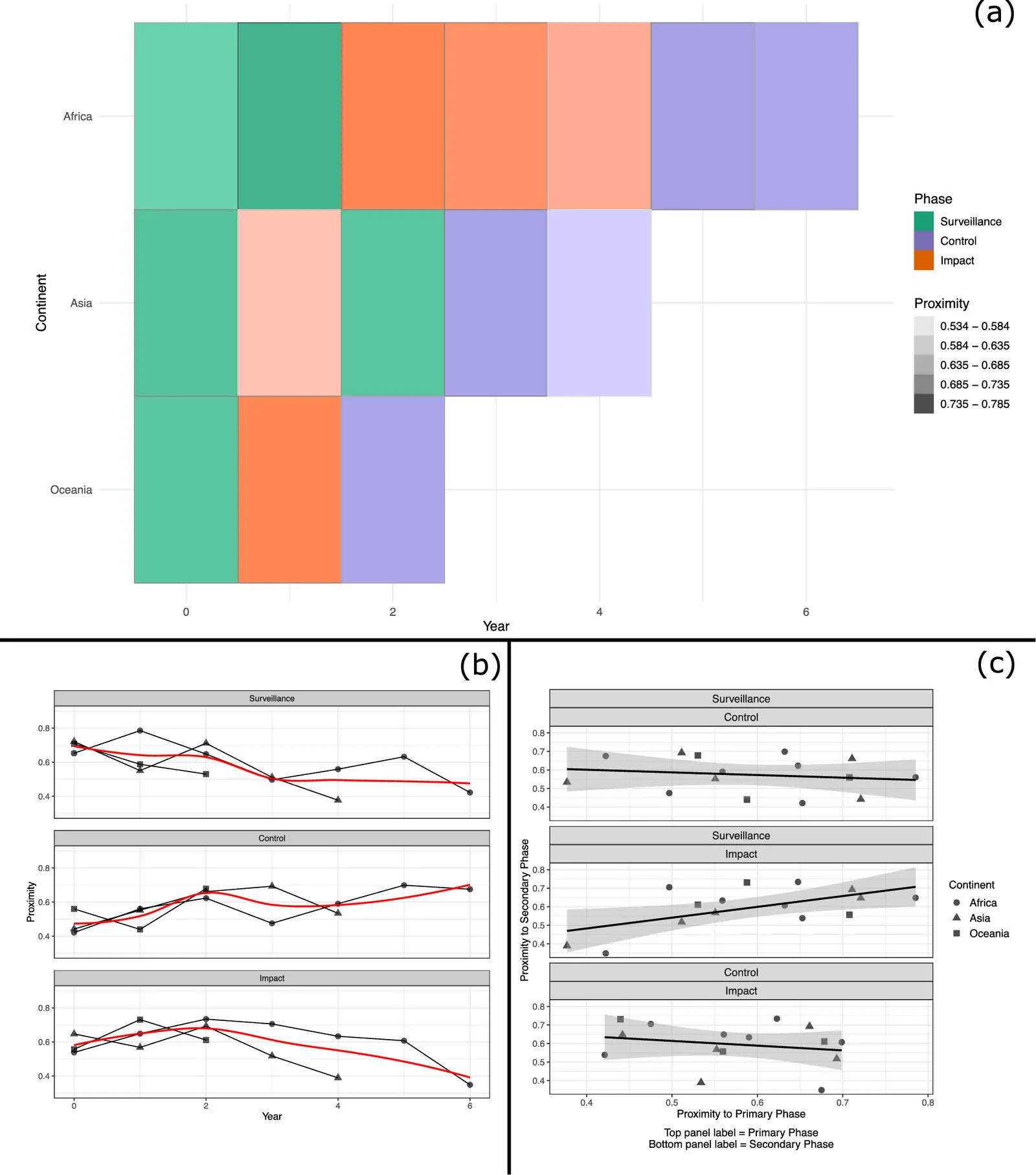
**a** Best-fitting biological invasion phase over time by continent based on thematic prevalence in news coverage. Each tile represents a year-continent combination and is colored by the phase (Surveillance, Control, or Impact) whose predicted theme profile most closely matched the observed distribution of themes in the news data for that region and year. The matching was based on the minimum mean absolute distance between observed normalized theme values and predicted phase profiles. Tile shading indicates the proximity of the match, with darker shades representing closer alignment between observed and predicted values. **b** Temporal trends in the proximity of thematic prevalence in news coverage to each phase by continent. **c** Continent-year observations plotted by proximity to pairs of phases

To assess how closely observed themes aligned with predicted theme-phase patterns, we classified each observed region-year pairing into a corresponding biological invasion phase. First, we normalized the observed theme proportions by rescaling them within each region-theme group to their local maxima. For each region and year, we then calculated the mean absolute distance between the observed normalized and predicted theme values. We assigned each observation to the phase with the minimum distance (Fig. 4).

## Results

### Overall temporal thematic trends

Over the duration of the invasion, and despite the length of this period varying between continents, we find similar patterns in temporal trends of themes (Fig. 3). Overall themes show temporal patterns that in part confirm the predictions of our idealized model combining expected news themes with invasion phases (Fig. 2). Confirming our prediction, all continents see an early emphasis on ‘Surveillance and Prevention’ (Africa = 0.387, Asia = 0.259, and Oceania = 0.722 in year 0) with one or both of the two control-oriented themes ‘Conventional Control’ (Africa = 0.228 in year 6, Asia = 0.452 in year 4, and Oceania = 0.109 in year 2), ‘Agroecological Control’ (Africa = 0.283 in year 6), and the ‘Research and Development’ theme (Africa = 0.307 in year 6 and Oceania = 0.296 in year 2) peaking later (Fig. 3). In contrast to our predictions, ‘Crop Impacts’ and ‘Social Impacts’ do not show a tendency for higher prevalence later in an invasion sequence, but in general occur in parallel to the control and ‘Research and Development’ themes, mentioned above. Among the themes that we predicted to occur across phases, the ‘Government’ theme confirms this pattern, while the ‘Agriculture’ and ‘Invasive Species’ themes occurred too rarely to find any conclusive patterns (Fig. 3).

### Thematic variation between continents

Across continents, there is variation in the prominence of themes. Africa features a thematic emphasis on both control types ‘Conventional Control’ (0.283 and 0.228 in years 5 and 6) and ‘Agroecological Control’ (0.283 in year 6), as well as ‘Surveillance and Prevention’ (0.387 and 0.315 in years 0 and 1), ‘Research and Development’ (0.307 in year 6), and ‘Government’ (0.205 and 0.160 in years 0 and 1) (Fig. 3). ‘Social Impacts’ (0.294, 0.205, and 0.212 in years 1, 3 and 4), ‘Conventional Control’ (0.270 and 0.452 in years 3 and 4), ‘Agroecological Control’ (ranges from 0.106 to 0.137 in years 1–4), and ‘Government’ (0.117 in year 3) are most prevalent in Asia’s discourse. However, the continent exhibits greater thematic breadth and less depth relative to other continents. For Oceania, ‘Surveillance and Prevention’ (0.722 in year 0) along with ‘Crop Impacts’ (0.529 in year 1) dominate both its intra- and inter-continental discourse (Fig. 3).

### Progression of invasion phases by region

Consistent with our idealized model, all three continents exhibit early emphasis on a Surveillance phase (Africa = 0.653, Asia = 0.721, and Oceania = 0.709 proximity in year 0) (Fig. 4). A progression from a Surveillance phase to an Impact one (Africa = range of 0.633–0.734 in years 2–4, Asia = 0.568 in year 1, Oceania = 0.731 in year 1), followed by a Control phase (Africa = 0.301 and 0.325 in years 5 and 6, Asia = 0.693 and 0.534 in years 3 and 4, Oceania = 0.678 in year 2), is evident across regions (Fig. 4).

## Discussion

### Media theme patterns in relation to invasion model

The observed phase trends may reflect an interplay between real-world invasion dynamics and media production practices. The progression of phases partly aligns with our idealized model predictions, particularly regarding an early Surveillance phase. However, the subsequent emergence of an Impact phase followed by a Control phase may indicate that consequences of the invasion unfolded rapidly and that control efforts were reactive, that early coverage included anticipated outcomes, or that it relied on anecdotal evidence and preliminary data. Alternatively, media outlets initially emphasized these topics, but attention subsequently declined following the issue-attention cycle, or the outbreak itself was effectively controlled.

### Interpreting continental thematic variation

Continental variations in these media representations of fall armyworm invasions appear to reflect the actual conditions and responses occurring within these regions. For Oceania, the prominence of the theme ‘Surveillance & Prevention’ aligns with the region’s demonstrated biosecurity measures and infrastructure. Australia and New Zealand, in particular, have rigorous biosecurity protocols and well-established institutions stemming from their need to protect their unique, geographically isolated ecosystems and significant agricultural sectors from invasive species. These measures are often regarded as more stringent than those of many other countries (Stone 2021). Additionally, the earlier outbreaks of FAW in Africa and Asia may have provided countries in Oceania advance warning, potentially increasing regional discussions around prevention and preparedness.

Both pest control methods themes are more predominantly emphasized in the discourse surrounding Africa and Asia. In Africa, prior to the invasion of FAW, the use of chemical insecticides among maize producers was minimal (Kenis et al. 2023). Post-invasion, synthetic pesticides have become the most common management method for FAW in Africa (Niassy et al. 2021). The government of Ghana, for instance, after having suffered financial losses, reacted by widely deploying insecticides (Safo et al. 2023). Additionally, the adoption of genetically modified crops on the African continent expanded beyond South Africa with the introduction of programs such as TELA maize in Nigeria (Orchardson 2024). Similarly, in China, the invasion has led to increased pesticide expenditure among smallholder farmers, with local maize growers in Yunnan primarily relying on pesticides for FAW management (Yang et al. 2021). In a survey conducted involving farmers from several South Asian countries, respondents reported using an assortment of management practices, with chemical pesticides being the dominant strategy (Khan et al. 2023). The theme of ‘Agroecological Control’ also emerges in the discourse of these regions, potentially driven by development efforts. For example, following the FAW invasion in Sub-Saharan Africa, governments and international and non-governmental organizations discussed training farmers and agricultural stakeholders on constructing integrated pest management (IPM) approaches for FAW control and management (Matova et al. 2020).

With regard to impact types, ‘Social Impacts’ proves more pronounced than ‘Crop Impacts’ in Africa and Asia, where subsistence farming is widespread and harm to crops has more direct social consequences (Giller et al. 2021). In both regions, FAW has disrupted smallholder farming systems, exacerbated household hunger, reduced incomes, and created other negative impacts on human health and the environment (Midega et al. 2023).

### Limitations of data sources and methods

An important consideration in this study is the extent to which the captured themes and their temporal patterns can be interpreted as cascading impacts. The captured themes do not necessarily reflect the exact reactions on the ground or measures being implemented. Instead, they indicate the importance or focus placed on these themes by media outlets. In the early stages of invasion, this focus may include concerns about potential or anticipated impacts that have not yet materialized. As such, some themes that appear early in the invasion timeline may reflect expectations about future consequences, rather than observed outcomes. The overall global trends are also likely to be heavily influenced by countries or regions with a larger number of articles available in English. To address this limitation, future work could incorporate news articles in additional languages, enabling a more balanced representation of regions where English-language coverage is limited. Expanding multilingual scraping and translation would help address current biases, strengthen global trend comparisons, and potentially facilitate finer-grained regional analysis.

Additionally, a theme may signify different practices or strategies depending on governmental, institutional, or geographical contexts. Although we identified overarching themes within regional discourse, not all countries are proportionally represented in available news articles, so country-level variation could not be assessed. The more recent invasion of Asia and Oceania also affects the quantity of available news coverage, as well as availability of FAW reporting data underlining assessment of impacts. This is particularly the case for China, Taiwan, India and South-East Asian countries, where documentation of yield losses due to FAW is limited (Overton et al. 2021).

Another challenge to identifying cascading impacts triggered by the introduction of FAW or other pests is that concerns about market access may incentivize countries to delay or avoid reporting invasive species. In Asia there have been reporting delays and inconsistencies that are known to have affected the perceived invasion pattern of FAW, supporting the “out of Africa” thesis (Kenis et al. 2023). Future studies could address this limitation by incorporating measures of overall media output, allowing for analyses to account for baseline reporting intensity across countries.

Lastly, although manual screening was used to remove clear false positives, keyword-based searches may still capture occasional articles in which fall armyworm was reported based on provisional or uncertain identification, or cases that were later corrected as another species. Conversely, the approach may also have excluded relevant articles that referenced fall armyworm using alternative terminology. Future research could incorporate common names and known synonyms to improve coverage.

### Extending news-driven frameworks to cascading impacts

Given that several temporal and regional thematic trends correspond to an existing biological invasion framework and real-world phenomena, this analytical approach demonstrates potential for identifying cascading impacts. This capability is increasingly needed, particularly considering the ongoing migration of FAW from North Africa toward Southern Europe, a process already documented by recent research (Wang et al. 2023; Kartakis et al. 2025). Utilizing structural topic modeling or comparable analytical methods focused on news media could provide timely monitoring of this evolving threat in Europe, thus supporting improved response strategies. Existing media-analysis tools such as PADI-web (Platform for Automated extraction of Disease Information from the web) could be broadened beyond their current focus on animal and plant pathogens to track other pest species, while also being expanded methodologically to capture downstream impacts (Valentin et al. 2021; Roche et al. 2024).

However, the current study is limited in its scope due to the focus on a single pest species and English-language sources. Future research should look to incorporate a wider range of pest species and a multilingual dataset to strengthen the robustness and applicability of the findings. Expanding the corpus to include more locally based news coverage would further support finer-grained spatial analysis. Additionally, future studies could conduct a form of linguistic analysis, such as examining verb tense, to distinguish between projected and reported events in the text, facilitating the exploration of actual cascading effects. This approach would make it possible to examine how an initial shock, such as crop loss, cascades into wider societal impacts, revealing the sequence and timing of these connections, as well as factors that drive or obstruct effective responses. Over longer time periods, this approach could also help assess whether biological invasions primarily generate short-term crisis responses or contribute to more enduring changes in governance, biosecurity, or agricultural systems. Understanding these dynamics, and the conditions that shape societal responses, can help reveal the capacities needed to navigate these disruptions more effectively.

## Conclusions

Structural topic model (STM) analysis reveals regional variations in popular news media discourse surrounding the FAW invasion, highlighting the influence of geographical context on the thematic evolution of FAW-related narratives. In particular, in Oceania, the continent where the FAW invasion is more recent, the discourse focuses more on preparatory actions compared to Africa and Asia. Additionally, all regions follow a broadly similar progression of biological invasion phases: Surveillance, Impact, and Control. Africa features a longer and proportionally more substantial Impact phase, providing insight into the cascading dynamics of such events. When combined with other data sources, findings from structural topic modeling of news media can contribute to a broader understanding of the cascading impacts of emerging agricultural pests.

Understanding these regional differences in discourse, and the underlying social-ecological impacts they reflect, is necessary for developing targeted and effective strategies to manage the consequences of FAW invasions. This need is particularly pressing as FAW has the potential to establish overwintering populations in Southern Europe. Applying STM or similar news media analysis methods to monitor evolving discourse in Europe could enhance preparedness and inform timely responses to this growing threat.

## Supplementary Information

Below is the link to the electronic supplementary material.Supplementary file1 (PDF 734 KB)

## Data Availability

This study is based on text and data mining of lawfully accessed news articles for non-commercial academic research. We do not redistribute the full texts of the articles, as these remain subject to copyright. The document-feature matrix, article metadata, and code to reproduce the dataset, preprocessing steps, and STM analysis are available at https://github.com/kathrynbjorklund/tracking-faw-invasion-phases-via-news-media.
