# Supplementary material for: Tracking phases of the fall armyworm (Spodoptera frugiperda) invasion across multiple continents using news media
Source: Biol Invasions. 2026 Jul 20;28(8):176. doi: 10.1007/s10530-026-03887-3 (PMC13385020; doi:10.1007/s10530-026-03887-3)
Supplement: Supplementary file 1 — Supplementary file1 (PDF 734 KB) [file 10530_2026_3887_MOESM1_ESM.pdf]

Supplementary Material

Tracking phases of the fall armyworm (*Spodoptera frugiperda*) invasion across multiple continents using news media

Kathryn Bjorklund[1], Melissa A. Barton[1], Stefan Daume[1,2], and Peter Søgaard Jørgensen[1,3]

[1] Stockholm Resilience Centre, Stockholm University, Stockholm, Sweden

[2] Beijer Institute for Ecological Economics, The Royal Swedish Academy of Sciences, Stockholm, Sweden

[3] Global Economic Dynamics and the Biosphere, The Royal Swedish Academy of Sciences, Stockholm, Sweden

* Correspondence to kathryn.bjorklund@su.se

Table of Contents

Appendix 1. References documenting the timeline of fall armyworm detection by country or territory ...2

Appendix 2. STM diagnostics with a wide range of K values 10

Appendix 3. Topic descriptors from STM with K = 33 11

Appendix 4. Theme creation process details 12

Appendix 5. Description of themes and topics 13

Appendix 6. Number of articles for which each topic was the most prevalent topic 15

Appendix 7. STM prevalence regression coefficients 16

Appendix 1. References documenting the timeline of fall armyworm detection by country or territory

Table A1 Year of fall armyworm detection by country or territory

Country/Territory	Year	Month if Confirmed	Notes	Reference
Nigeria	2016	January		Goergen G, Kumar PL, Sankung SB, et al (2016) First Report of Outbreaks of the Fall Armyworm Spodoptera frugiperda (J E Smith) (Lepidoptera, Noctuidae), a New Alien Invasive Pest in West and Central Africa. PLoS One 11:e0165632. https://doi.org/10.1371/journal.pone.0165632
Benin	2016		Later in 2016	Goergen G, Kumar PL, Sankung SB, et al (2016) First Report of Outbreaks of the Fall Armyworm Spodoptera frugiperda (J E Smith) (Lepidoptera, Noctuidae), a New Alien Invasive Pest in West and Central Africa. PLoS One 11:e0165632. https://doi.org/10.1371/journal.pone.0165632
Togo	2016		Later in 2016	Goergen G, Kumar PL, Sankung SB, et al (2016) First Report of Outbreaks of the Fall Armyworm Spodoptera frugiperda (J E Smith) (Lepidoptera, Noctuidae), a New Alien Invasive Pest in West and Central Africa. PLoS One 11:e0165632. https://doi.org/10.1371/journal.pone.0165632
São Tomé and Príncipe	2016	April		Goergen G, Kumar PL, Sankung SB, et al (2016) First Report of Outbreaks of the Fall Armyworm Spodoptera frugiperda (J E Smith) (Lepidoptera, Noctuidae), a New Alien Invasive Pest in West and Central Africa. PLoS One 11:e0165632. https://doi.org/10.1371/journal.pone.0165632
Gabon	2016			Goergen G, Kumar PL, Sankung SB, et al (2016) First Report of Outbreaks of the Fall Armyworm Spodoptera frugiperda (J E Smith) (Lepidoptera, Noctuidae), a New Alien Invasive Pest in West and Central Africa. PLoS One 11:e0165632. https://doi.org/10.1371/journal.pone.0165632
Democratic Republic of the Congo	2017	April		Goergen G, Kumar PL, Sankung SB, et al (2016) First Report of Outbreaks of the Fall Armyworm Spodoptera frugiperda (J E Smith) (Lepidoptera, Noctuidae), a New Alien Invasive Pest in West and Central Africa. PLoS One 11:e0165632. https://doi.org/10.1371/journal.pone.0165632
Eswatini	2017	April		Goergen G, Kumar PL, Sankung SB, et al (2016) First Report of Outbreaks of the Fall Armyworm Spodoptera frugiperda (J E Smith) (Lepidoptera, Noctuidae), a New Alien Invasive Pest in West and Central Africa. PLoS One 11:e0165632. https://doi.org/10.1371/journal.pone.0165632

Ghana	2017	April		Goergen G, Kumar PL, Sankung SB, et al (2016) First Report of Outbreaks of the Fall Armyworm Spodoptera frugiperda (J E Smith) (Lepidoptera, Noctuidae), a New Alien Invasive Pest in West and Central Africa. PLoS One 11:e0165632. https://doi.org/10.1371/journal.pone.0165632
Mozambique	2017	April		Goergen G, Kumar PL, Sankung SB, et al (2016) First Report of Outbreaks of the Fall Armyworm Spodoptera frugiperda (J E Smith) (Lepidoptera, Noctuidae), a New Alien Invasive Pest in West and Central Africa. PLoS One 11:e0165632. https://doi.org/10.1371/journal.pone.0165632
South Africa	2017	April		Goergen G, Kumar PL, Sankung SB, et al (2016) First Report of Outbreaks of the Fall Armyworm Spodoptera frugiperda (J E Smith) (Lepidoptera, Noctuidae), a New Alien Invasive Pest in West and Central Africa. PLoS One 11:e0165632. https://doi.org/10.1371/journal.pone.0165632
Zambia	2017	April		Goergen G, Kumar PL, Sankung SB, et al (2016) First Report of Outbreaks of the Fall Armyworm Spodoptera frugiperda (J E Smith) (Lepidoptera, Noctuidae), a New Alien Invasive Pest in West and Central Africa. PLoS One 11:e0165632. https://doi.org/10.1371/journal.pone.0165632
Zimbabwe	2017	April		Goergen G, Kumar PL, Sankung SB, et al (2016) First Report of Outbreaks of the Fall Armyworm Spodoptera frugiperda (J E Smith) (Lepidoptera, Noctuidae), a New Alien Invasive Pest in West and Central Africa. PLoS One 11:e0165632. https://doi.org/10.1371/journal.pone.0165632
Mali	2017	July		Day R, Abrahams P, Bateman M, et al (2017) Fall Armyworm: Impacts and Implications for Africa. Outlooks on Pest Management 28:196–201. https://doi.org/10.1564/v28_oct_02
Angola	2017	August		Day R, Abrahams P, Bateman M, et al (2017) Fall Armyworm: Impacts and Implications for Africa. Outlooks on Pest Management 28:196–201. https://doi.org/10.1564/v28_oct_02
Botswana	2017	August		Day R, Abrahams P, Bateman M, et al (2017) Fall Armyworm: Impacts and Implications for Africa. Outlooks on Pest Management 28:196–201. https://doi.org/10.1564/v28_oct_02
Burkina Faso	2017	August		Day R, Abrahams P, Bateman M, et al (2017) Fall Armyworm: Impacts and Implications for Africa. Outlooks on Pest Management 28:196–201. https://doi.org/10.1564/v28_oct_02
Burundi	2017	August		Day R, Abrahams P, Bateman M, et al (2017) Fall Armyworm: Impacts and Implications for Africa. Outlooks on Pest Management 28:196–201. https://doi.org/10.1564/v28_oct_02

Cameroon	2017	August		Day R, Abrahams P, Bateman M, et al (2017) Fall Armyworm: Impacts and Implications for Africa. Outlooks on Pest Management 28:196–201. https://doi.org/10.1564/v28_oct_02
Chad	2017	August		Day R, Abrahams P, Bateman M, et al (2017) Fall Armyworm: Impacts and Implications for Africa. Outlooks on Pest Management 28:196–201. https://doi.org/10.1564/v28_oct_02
Ethiopia	2017	August		Day R, Abrahams P, Bateman M, et al (2017) Fall Armyworm: Impacts and Implications for Africa. Outlooks on Pest Management 28:196–201. https://doi.org/10.1564/v28_oct_02
Guinea	2017	August		Day R, Abrahams P, Bateman M, et al (2017) Fall Armyworm: Impacts and Implications for Africa. Outlooks on Pest Management 28:196–201. https://doi.org/10.1564/v28_oct_02
Malawi	2017	August		Day R, Abrahams P, Bateman M, et al (2017) Fall Armyworm: Impacts and Implications for Africa. Outlooks on Pest Management 28:196–201. https://doi.org/10.1564/v28_oct_02
Namibia	2017	August		Day R, Abrahams P, Bateman M, et al (2017) Fall Armyworm: Impacts and Implications for Africa. Outlooks on Pest Management 28:196–201. https://doi.org/10.1564/v28_oct_02
Niger	2017	August		Day R, Abrahams P, Bateman M, et al (2017) Fall Armyworm: Impacts and Implications for Africa. Outlooks on Pest Management 28:196–201. https://doi.org/10.1564/v28_oct_02
Republic of the Congo	2017	August		Day R, Abrahams P, Bateman M, et al (2017) Fall Armyworm: Impacts and Implications for Africa. Outlooks on Pest Management 28:196–201. https://doi.org/10.1564/v28_oct_02
Rwanda	2017	August		Day R, Abrahams P, Bateman M, et al (2017) Fall Armyworm: Impacts and Implications for Africa. Outlooks on Pest Management 28:196–201. https://doi.org/10.1564/v28_oct_02
South Sudan	2017	August		Day R, Abrahams P, Bateman M, et al (2017) Fall Armyworm: Impacts and Implications for Africa. Outlooks on Pest Management 28:196–201. https://doi.org/10.1564/v28_oct_02
Tanzania	2017	August		Day R, Abrahams P, Bateman M, et al (2017) Fall Armyworm: Impacts and Implications for Africa. Outlooks on Pest Management 28:196–201. https://doi.org/10.1564/v28_oct_02
Uganda	2017	August		Day R, Abrahams P, Bateman M, et al (2017) Fall Armyworm: Impacts and Implications for Africa. Outlooks on Pest Management 28:196–201. https://doi.org/10.1564/v28_oct_02
Gambia	2017	November		FAO (Food and Agriculture Organization) (2018) Briefing Note on FAO Actions on Fall Armyworm in Africa. FAO
Côte d'Ivoire	2017	November		FAO (Food and Agriculture Organization) (2018) Briefing Note on FAO Actions on Fall Armyworm in Africa. FAO

Cabo Verde	2017			FAO (Food and Agriculture Organization) (2017) Briefing Note on FAO Actions on Fall Armyworm in Africa. FAO, Rome, Italy
Central African Republic	2017			FAO (Food and Agriculture Organization) (2018) Briefing Note on FAO Actions on Fall Armyworm in Africa. FAO
Guinea-Bissau	2017			FAO (Food and Agriculture Organization) (2017) Briefing Note on FAO Actions on Fall Armyworm in Africa. FAO, Rome, Italy
Madagascar	2017			FAO (Food and Agriculture Organization) (2017) Briefing Note on FAO Actions on Fall Armyworm in Africa. FAO, Rome, Italy
Senegal	2017			FAO (Food and Agriculture Organization) (2017) Briefing Note on FAO Actions on Fall Armyworm in Africa. FAO, Rome, Italy
Seychelles	2017			FAO (Food and Agriculture Organization) (2017) Briefing Note on FAO Actions on Fall Armyworm in Africa. FAO, Rome, Italy
Sierra Leone	2017			FAO (Food and Agriculture Organization) (2017b) FAO Advisory Note on Fall Armyworm (FAW) in Africa. FAO, Rome, Italy
Somalia	2017			FAO (Food and Agriculture Organization) (2017) Briefing Note on FAO Actions on Fall Armyworm in Africa. FAO, Rome, Italy
Sudan	2017			Day R, Abrahams P, Bateman M, et al (2017) Fall Armyworm: Impacts and Implications for Africa. Outlooks on Pest Management 28:196–201. https://doi.org/10.1564/v28_oct_02
Liberia	2018	February		FAO (Food and Agriculture Organization) (2018) Briefing Note on FAO Actions on Fall Armyworm in Africa. FAO
Eritrea	2018	July		IPPC (International Plant Protection Convention) (2018a) Eritrea: Crops are safe from Fall Armyworm infestation. Ministry of Agriculture of The State of Eritrea
India<br>- Andhra Pradesh<br>- Arunachal Pradesh<br>- Assam<br>- Bihar<br>- Chhattisgarh<br>- Goa<br>- Gujarat<br>- Himachal Pradesh<br>- Jharkhand<br>- Karnataka<br>- Kerala<br>- Madhya Pradesh<br>- Maharashtra<br>- Manipur<br>- Meghalaya	2018			ICAR-NBAIR (Indian Council of Agricultural Research - National Bureau of Agricultural Insect Resources) (2018) PEST ALERT: Spodoptera frugiperda (Smith, J.E.) (Insecta: Lepidoptera). ICAR-NBAIR, India

- Mizoram<br>- Nagaland<br>- Odisha<br>- Punjab<br>- Rajasthan<br>- Sikkim<br>- Tamil Nadu<br>- Telangana<br>- Tripura<br>- Uttar Pradesh<br>- Uttarakhand<br>- West Bengal				
Thailand	2018			IPPC (International Plant Protection Convention) (2018b) First detection of Fall Army Worm on the border of Thailand
Mauritius	2019	March		MSIRI (Mauritius Sugarcane Industry Research Institute) (2019) Fall Armyworm Observed in Mauritius. Mauritius Sugarcane Industry Research Institute, Mauritius
Nepal	2019	May		Adhikari K, Bhandari S, Dhakal L, Shrestha J (2020) Fall armyworm (Spodoptera frugiperda): A threat in crop production in Africa and Asia. Peruvian Journal of Agronomy 4:121–133. https://doi.org/10.21704/pja.v4i3.1495
Taiwan	2019	June		Tsai C-L, Chu I-H, Chou M-H, et al (2020) Rapid identification of the invasive fall armyworm Spodoptera frugiperda (Lepidoptera, Noctuidae) using species-specific primers in multiplex PCR. Sci Rep 10:16508. https://doi.org/10.1038/s41598-020-73786-7
Bhutan	2019	September		Mahat K, Mitchell A, Zangpo T (2021) An updated global COI barcode reference data set for Fall Armyworm (*Spodoptera frugiperda*) and first record of this species in Bhutan. Journal of Asia-Pacific Entomology 24:105–109. https://doi.org/10.1016/j.aspen.2020.11.013
Egypt	2019	November		FAO (Food and Agriculture Organization) (2024) FAW Map, Global Action for Fall Armyworm Control. FAO
Yemen	2019	November		FAO (Food and Agriculture Organization) (2024) FAW Map, Global Action for Fall Armyworm Control. FAO
Bangladesh	2019	November		FAO (Food and Agriculture Organization) (2024) FAW Map, Global Action for Fall Armyworm Control. FAO
Cambodia	2019	November		FAO (Food and Agriculture Organization) (2024) FAW Map, Global Action for Fall Armyworm Control. FAO
China<br>- Anhui<br>- Beijing	2019	November		FAO (Food and Agriculture Organization) (2024) FAW Map, Global Action for Fall Armyworm Control. FAO

- Chongqing<br>- Fujian<br>- Gansu<br>- Guangdong<br>- Guangxi<br>- Guizhou<br>- Hainan<br>- Hebei<br>- Henan<br>- Hubei<br>- Hunan<br>- Jiangsu<br>- Jiangxi<br>- Liaoning<br>- Ningxia<br>- Shaanxi<br>- Shandong<br>- Shanghai<br>- Shanxi<br>- Sichuan<br>- Yunnan<br>- Zhejiang<br>- Hong Kong				
Indonesia	2019	November		FAO (Food and Agriculture Organization) (2024) FAW Map, Global Action for Fall Armyworm Control. FAO
Japan	2019	November		FAO (Food and Agriculture Organization) (2024) FAW Map, Global Action for Fall Armyworm Control. FAO
Laos	2019	November		FAO (Food and Agriculture Organization) (2024) FAW Map, Global Action for Fall Armyworm Control. FAO
Malaysia	2019	November		FAO (Food and Agriculture Organization) (2024) FAW Map, Global Action for Fall Armyworm Control. FAO
Myanmar	2019	November		FAO (Food and Agriculture Organization) (2024) FAW Map, Global Action for Fall Armyworm Control. FAO
Philippines	2019	November		FAO (Food and Agriculture Organization) (2024) FAW Map, Global Action for Fall Armyworm Control. FAO
South Korea	2019	November		FAO (Food and Agriculture Organization) (2024) FAW Map, Global Action for Fall Armyworm Control. FAO
Sri Lanka	2019	November		FAO (Food and Agriculture Organization) (2024) FAW Map, Global Action for Fall Armyworm Control. FAO

Vietnam	2019	November		FAO (Food and Agriculture Organization) (2024) FAW Map, Global Action for Fall Armyworm Control. FAO
Pakistan	2019	November		Khan HA, Ali N, Farooq MU, Gill NA, Ahmad T, Khalique U (2020) First authentic report of fall armyworm presence in Faisalabad, Pakistan. *J Entomol Zool Stud* 8(4):1512–1514
Brunei	2019	November		Association of Southeast Asian Nations (2021) ASEAN Action Plan on Fall Armyworm Control. Association of Southeast Asian Nations
New Caledonia (FR)	2020	January		FAO (Food and Agriculture Organization) (2024) FAW Map, Global Action for Fall Armyworm Control. FAO
Australia<br>- New South Wales<br>- Northern Territory<br>- Queensland<br>- Tasmania<br>- Victoria<br>- Western Australia	2020	Between February and May		FAO (Food and Agriculture Organization) (2024) FAW Map, Global Action for Fall Armyworm Control. FAO
Mauritania	2020	Between February and May		FAO (Food and Agriculture Organization) (2024) FAW Map, Global Action for Fall Armyworm Control. FAO
Timor-Leste	2020	Between February and May		FAO (Food and Agriculture Organization) (2024) FAW Map, Global Action for Fall Armyworm Control. FAO
United Arab Emirates	2020	Between February and May		FAO (Food and Agriculture Organization) (2024) FAW Map, Global Action for Fall Armyworm Control. FAO
Israel	2020	June		EPPO (European and Mediterranean Plant Protection Organization) (2020) First Report of Spodoptera frugiperda in Israel. EPPO
Jordan	2020		Late 2020	FAO (Food and Agriculture Organization) (2024) FAW Map, Global Action for Fall Armyworm Control. FAO
Syria	2020		Late 2020	FAO (Food and Agriculture Organization) (2024) FAW Map, Global Action for Fall Armyworm Control. FAO
Papua New Guinea	2020		Late 2020	FAO (Food and Agriculture Organization) (2024) FAW Map, Global Action for Fall Armyworm Control. FAO
Canary Islands (ES)	2021	April		FAO (Food and Agriculture Organization) (2024) FAW Map, Global Action for Fall Armyworm Control. FAO
Solomon Islands (UK)	2021	September		IPPC (International Plant Protection Convention) (2021) Fall Armyworm has been detected in Solomon Island
Saudi Arabia	2021	October		IPPC (International Plant Convention) (2022) Fall armyworm detected in the Kingdom of Saudi Arabia
Norfolk Island (AU)	2021			IPPC (International Plant Protection Convention) (2021) Spodoptera frugiperda (fall

				armyworm) detections Australia. FAO (Food and Agriculture Organization), Rome, Italy
Tasmania (AU)	2021			IPPC (International Plant Protection Convention) (2021) Spodoptera frugiperda (fall armyworm) detections Australia. FAO (Food and Agriculture Organization), Rome, Italy
New Zealand	2022	March		IPPC (International Plant Protection Convention) (2022) New Zealand, Pest Reports, Spodoptera frugiperda (Fall armyworm)
Comoros	2022			EPPO (European and Mediterranean Plant Protection Organization) (2023) EPPO Global Database
Turkey	2022			PehliVan S (2022) First record of the fall armyworm, Spodoptera frugiperda (J.E. Smith, 1797) (Lepidoptera: Noctuidae) in Türkiye. CJAFS 37. https://doi.org/10.36846/CJAFS.2022.82
Cyprus	2023			EPPO (European and Mediterranean Plant Protection Organization) (2023) EPPO Global Database
Greece	2023			EPPO (European and Mediterranean Plant Protection Organization) (2023) EPPO Global Database
Portugal	2023			EPPO (European and Mediterranean Plant Protection Organization) (2023) EPPO Global Database
Romania	2023			EPPO (European and Mediterranean Plant Protection Organization) (2023) EPPO Global Database
Vanuatu	2023	June		IPPC (International Plant Protection Convention) (2024) Vanuatu, Pest Reports, Spodoptera frugiperda (Fall armyworm)
Malta	2023	September		Seguna A, Catania A, Borg JJ, Sammut P (2024) Spodoptera frugiperda (Smith, 1797), an unwelcome visitor reaches the Maltese Islands (Lepidoptera: Noctuidae, Xyleninae). SHILAP Revta lepid 52:29–31. https://doi.org/10.57065/shilap.844
Iran	2023	October		EPPO (European and Mediterranean Plant Protection Organization) (2024) First Report of Spodoptera frugiperda in Iran. EPPO

Appendix 2. STM diagnostics with a wide range of K values

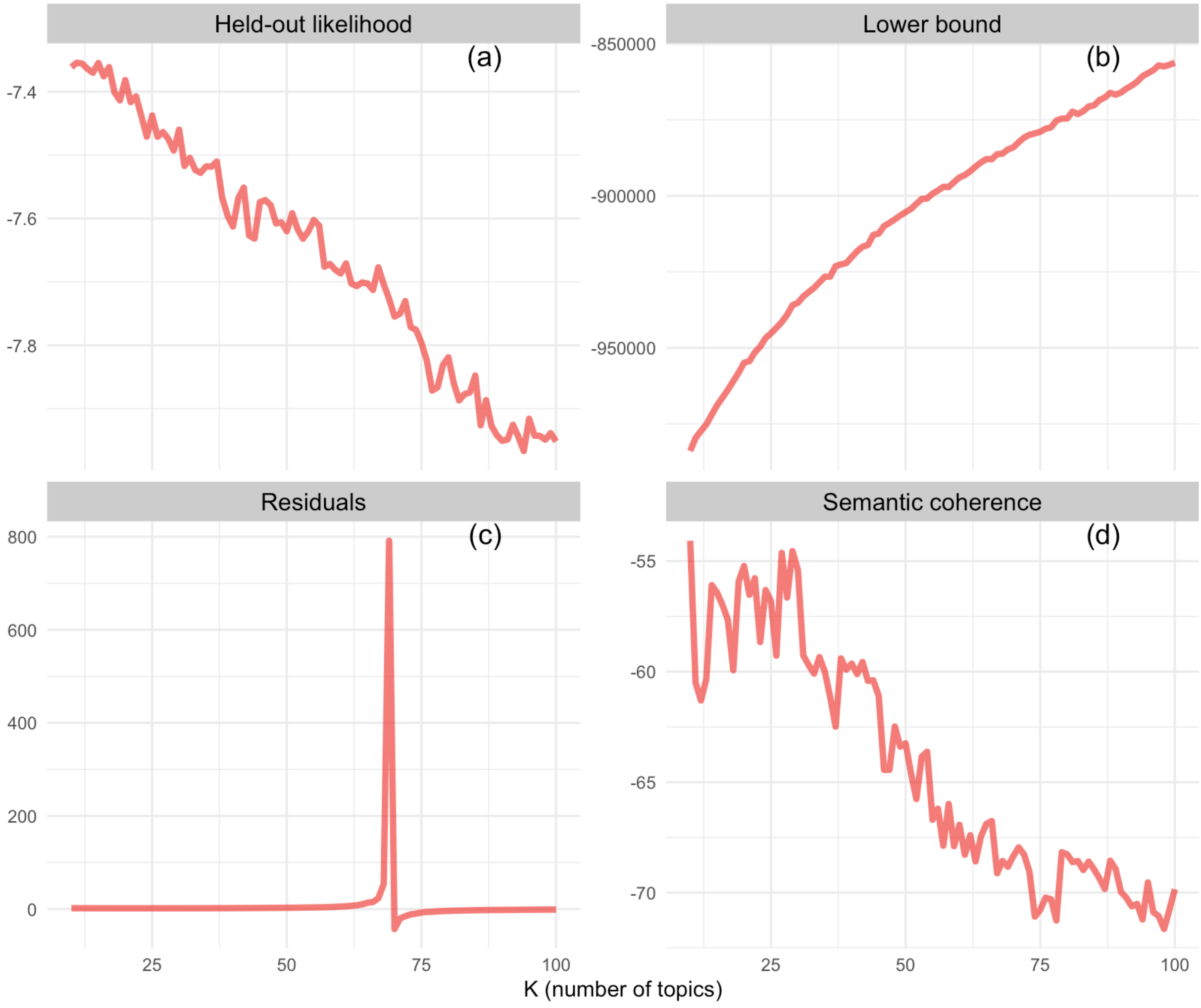

Fig. A2 Topics 10-100 incremented by 1 (a) Held-out likelihood decreases from K = 10 and steadies at K = 32, (b) lower bound increases logarithmically from K = 10, (c) residuals increase gradually until K = 69 before sharply increasing, (d) semantic coherence decreases from K = 30

Appendix 3. Topic descriptors from STM with K = 33

Table A3 STM K = 33 high probability (Prob) and frequent and exclusive (FREX) words

Topic #	Prob	FREX
1	ravag, spread, studi, attack, global, risk, first	ravag, iboh, kgs, paper, litr, banana, travel
2	fao, food, system, locust, manag, develop, water	locust, guid, event, upadhya, swarm, flood, fao
3	adult, maculosus, oak, liaon, provinc, stage, overwint	maculosus, oak, liaon, overwint, rhynchaenus, blotch, reform
4	offici, rice, world, product, ministri, year, govern	cocacola, swire, dppqs, rumduol, sweetcorn, khmer, nov
5	say, district, garden, destroy, acr, spray, villag	cue, garden, plenti, subcounti, bong, mpi, acr
6	food, drought, region, peopl, year, report, southern	grand, sud, insecur, drought, humanitarian, crisi, conflict
7	invas, attack, pesticid, govern, countri, food, invad	invas, invad, entir, happen, cross, attack, kill
8	programm, region, plot, women, input, farm, pfumvudza	intwasa, pfumvudza, women, mrs, programm, input, plot
9	armi, general, control, ministri, director, report, depart	armi, compens, deleg, general, suspect, daff, held
10	spinetoram, control, damag, seed, treatment, infest, sfb	sfb, greas, spinetoram, seedpellet, halflif, cabbag, treatment
11	human, full, come, product, report, area, countri	multitud, full, download, curm, maliza, miracul, welloil
12	control, manag, research, pesticid, effect, ipm, intern	ipm, bcas, model, tambo, biopesticid, mon, campaign
13	destroy, pesticid, control, counti, affect, use, spread	blaster, standard, counti, multimedia, equatori, pepper, municip
14	use, control, biolog, insecticid, remus, parasitoid, keni	keni, remus, telenomus, parasitoid, reserv, sickl, cell
15	onion, import, water, increas, price, food, fish	onion, fish, rupe, kilo, retail, tuna, debt
16	spread, first, feed, report, outbreak, damag, region	ramasami, walsh, rapid, subtrop, telegraph, websit, biosecur
17	chang, climat, control, increas, natur, research, use	chang, climat, temperatur, elev, co2, household, enemi
18	grain, control, natur, grower, high, product, year	fawligen, grdc, spafford, grower, grub, benefici, grain
19	address, purpos, form, govern, report, food, content	phys, recipi, publish, purpos, request, content, address
20	state, district, attack, infest, year, damag, report	mizoram, mween, bastar, kvk, kharif, district, sow
21	pesticid, affect, district, ministri, area, outbreak, hectar	procur, ministri, district, outbreak, litr, minist, affect
22	product, seed, compani, varieti, research, hybrid, cimmyt	hybrid, cimmyt, iar, trial, toler, compani, approv
23	app, nuru, fao, help, launch, develop, research	nuru, app, satellit, pushpul, languag, launch, famew
24	region, infest, manag, citi, provinc, rice, control	philric, citi, da, task, asean, rcpc, forc
25	use, control, leav, effect, method, egg, general	assin, shred, doa, tree, soldier, garlic, residu
26	spread, report, first, cotton, damag, research, state	beher, litchi, guava, borer, babar, trunk, cotton
27	depart, control, attack, quarantin, damag, first, fli	quarantin, pressler, odisha, mile, papaya, mealybug, airport
28	destruct, found, biosecur, industri, spread, work, grower	destruct, biosecur, strait, torr, island, nt, lett
29	research, product, food, technolog, health, develop, use	apiacea, gmos, greenlight, technolog, safeti, caa, gene
30	food, fao, nation, spread, secur, govern, countri	fao, budget, gender, wfp, biosecur, mainstream, hpai
31	arm, say, agroecolog, seed, nanjundaswami, pesticid, mongabay	nanjundaswami, chukki, krrs, cargil, mongabay, agroecolog, reclaim
32	chemic, scientist, trap, control, includ, spread, pesticid	icar, bird, sugarcan, grass, syngenta, pherom, attract
33	year, food, pesticid, spread, say, fao, control	stapl, fao, alreadi, threaten, fear, told, say

Appendix 4. Theme creation process details

We created the nine themes through an iterative, collaborative process grounded in both the data and the literature. Prior to grouping, we had developed familiarity with the corpus contents through manual review of each article during region identification and inspection of the five most representative documents per topic during topic labeling. All authors contributed to the grouping exercise, drawing on collective expertise in emerging pests and pathogens, invasion biology, and impact cascades. We used the Welsh et al. (2021) biological invasion framework as a key conceptual structure for grouping. We grouped topics according to their semantics within the broader conversation of understanding invasion phases, refining assignments across multiple iterations until reaching consensus.

Appendix 5. Description of themes and topics

Table A5 Themes with accompanying decriptions, associated topics, and topic descriptions

Theme	Theme Description	Topic	Topic Description
Agriculture	Impacts of FAW on crop production, farming systems and agricultural development	(5) Domestic Agriculture	Household-level production, small-scale agriculture
		(15) Agricultural Sector	Larger-scale information about crops and trade
Government	Role of local, national and international institutions in managing the impacts of FAW	(8) Agricultural Empowerment Programs	Efforts to strengthen the capacity of farmers and other stakeholders to manage the pest and build resilient agricultural systems; gender-sensitive programming
		(9) Financial Aid	Financial assistance provided to farmers and other stakeholders to mitigate the economic impacts of FAW
		(24) Local Government Involvement	Role of local governments in managing the pest and promoting sustainable agriculture at the community level
		(27) Agriculture Department Work	Technical, economic and political dimensions of managing FAW
		(30) National Government Budgeting and Programming	Role of national governments in funding and implementing FAW interventions
Invasive Species	Shifts in temperature, precipitation and other environmental factors in relation to phenology, range and population of invasive pests	(17) Climate and Invasive Pests	The effect of changes in temperature and precipitation on survival and reproduction of pest species
		(26) Invasive Pest Species	Information about different invasive pest species; the organisms themselves and their characteristics
Social Impacts	Impacts of FAW on agricultural workers and communities at large	(2) Socioeconomic Impacts	Reduced income for farmers, discussion of potential for reduction in the availability of staple foods for different populations
		(7) Food Insecurity	Impact of FAW on food crops and feeding populations
		(20) Farmer Burdens	Burdens placed on individual farmers, household budgets
Crop Impacts	Impacts of FAW on physical plants and crop counts	(4) Crop Losses	Extent and distribution of crop losses caused by FAW
		(11) Damage	Descriptions of extent and type of damage caused to (primarily maize) plants
		(18) Productivity and Yields	Agricultural productivity measurements
Agroecological Control	Diversity, effectiveness and broader implications of agroecological pest management techniques	(13) Homemade Pest Control Remedies	Benefits and limitations of the use of natural methods; risks and hazards associated with them
		(14) Biological Pest Control Research	Development and testing the use of living organisms as pest control agents
		(19) Natural Enemies	Diversity and abundance of natural enemy species in different ecosystems, the factors that influence their effectiveness as pest control agents
		(25) Integrated Pest Management	Descriptions of different types of integrated pest management strategies that can be used to manage fall armyworm including the use of natural enemies, crop rotation, trap crops and other cultural practices

		(32) Agroecological Pest Control Methods	Principles of agroecology; different types of agroecological pest control methods that can be used to manage FAW, such as crop rotation, intercropping and cultural practices; broader implications of using agroecological pest control methods for agricultural workers, agriculture and the environment
Research & Development	Exploration in the ways in which technological innovations are transforming the field of pest management	(3) Invasive Pest Species Research	The scientific study of invasive species, including their ecology, distribution and impacts on native ecosystems
		(23) Technology	Use of phone applications, satellites, etc. in FAW detection and management
		(29) Research and Development/Innovation	Agriscience technologies, precision agriculture
Conventional Control	Diversity, effectiveness and broader implications of conventional pest management techniques	(10) Pesticide Research	Studies on development and effectiveness of new pesticides
		(12) Biotechnology/Gene Editing FAW	The genetic makeup of FAW and the potential targets for gene modification; implications of gene editing in addressing pest challenges
		(21) Pesticide Procurement	Details about the process of acquiring pesticides in different locations
		(22) Transgenic Crops/Genetically Modified Organisms	Different types of transgenic crops that have been developed or employed to manage FAW, including crops that produce insecticidal proteins or that are resistant to herbicides; studies of effectiveness of these transgenic crops in managing FAW
		(31) Seed Treatments	Use of chemical or biological agents to protect seeds and seedlings from pests and diseases
Surveillance & Prevention	Strategies and tools used to prevent the spread of FAW and detect its presence in agricultural settings	(1) Spread of the fall armyworm (FAW)	Biology and ecology of FAW, including its life cycle, behavior and distribution
		(6) Drought/Weather Patterns and FAW	The impacts of drought and weather patterns on the distribution and prevalence of FAW
		(16) Biosecurity	Border control, quarantine measures and surveillance
		(28) Insect Monitoring	Detection methods, e.g., pheromone traps
		(33) Invasion Preparation	Alerts about the potential threat of FAW, information about its spread in other countries

Appendix 6. Number of articles for which each topic was the most prevalent topic

Table A6 Topics with accompanying article number where it is most prevalent

Topic	Articles where topic is most prevalent, n	Percentage of corpus (%)
(20) Farmer Burdens	45	7.9
(21) Pesticide Procurement	36	6.3
(6) Drought/Weather Patterns and FAW	35	6.1
(30) National Government Budgeting and Programming	35	6.1
(24) Local Government Involvement	26	4.6
(28) Insect Monitoring	26	4.6
(29) Research and Development/Innovation	23	4
(5) Domestic Agriculture	21	3.7
(13) Homemade Pest Control Remedies	21	3.7
(33) Invasion Preparation	21	3.7
(12) Biotechnology/Gene Editing FAW	19	3.3
(26) Invasive Pest Species	19	3.3
(16) Biosecurity	17	3
(22) Transgenic Crops/Genetically Modified Organisms	17	3
(2) Socioeconomic Impacts	16	2.8
(4) Crop Losses	15	2.6
(9) Financial Aid	14	2.5
(11) Damage	14	2.5
(19) Natural Enemies	14	2.5
(17) Climate and Invasive Pests	13	2.3
(23) Technology	13	2.3
(14) Biological Pest Control Research	12	2.1
(15) Agricultural Sector	12	2.1
(18) Productivity and Yields	12	2.1
(1) Spread of the fall armyworm (FAW)	11	1.9
(8) Agricultural Empowerment Programs	11	1.9
(32) Agroecological Pest Control Methods	11	1.9
(27) Agriculture Department Work	9	1.6
(10) Pesticide Research	8	1.4
(25) Integrated Pest Management	8	1.4
(3) Invasive Pest Species Research	6	1.1
(7) Food Insecurity	5	0.9
(8) Seed Treatments	5	0.9

Appendix 7. STM prevalence regression coefficients

Table A7 STM prevalence regression coefficients by continent and invasion year

Topic #	Coefficient	Estimate	Std. error	t value	p value
**1**	(Intercept)	0.030	0.012	2.491	**0.013 ***
	Asia	0.013	0.019	0.699	**0.485**
	Oceania	−0.029	0.020	−1.453	**0.147**
	Invasion year	−0.005	0.003	−1.618	**0.106**
	Asia × invasion year	−0.002	0.007	−0.257	**0.797**
	Oceania × invasion year	0.005	0.019	0.258	**0.796**
**2**	(Intercept)	0.066	0.017	3.871	**< 0.001 *****
	Asia	−0.058	0.022	−2.688	**0.007 ****
	Oceania	−0.065	0.027	−2.401	**0.017 ***
	Invasion year	−0.008	0.005	−1.749	**0.081 .**
	Asia × invasion year	0.011	0.009	1.269	**0.205**
	Oceania × invasion year	0.009	0.026	0.347	**0.729**
**3**	(Intercept)	0.002	0.008	0.292	**0.771**
	Asia	0.004	0.012	0.289	**0.773**
	Oceania	0.000	0.015	−0.007	**0.994**
	Invasion year	0.003	0.003	1.228	**0.220**
	Asia × invasion year	0.001	0.006	0.088	**0.930**
	Oceania × invasion year	−0.004	0.015	−0.236	**0.813**
**4**	(Intercept)	0.031	0.013	2.277	**0.023 ***
	Asia	0.046	0.023	2.002	**0.046 ***
	Oceania	−0.030	0.024	−1.263	**0.207**
	Invasion year	−0.007	0.004	−1.674	**0.095 .**
	Asia × invasion year	−0.014	0.009	−1.586	**0.113**
	Oceania × invasion year	0.006	0.023	0.274	**0.784**
**5**	(Intercept)	0.013	0.014	0.874	**0.383**
	Asia	−0.011	0.020	−0.523	**0.601**
	Oceania	0.067	0.030	2.265	**0.024 ***
	Invasion year	0.011	0.005	2.354	**0.019 ***
	Asia × invasion year	−0.010	0.009	−1.178	**0.239**
	Oceania × invasion year	−0.043	0.032	−1.348	**0.178**
**6**	(Intercept)	0.114	0.021	5.423	**< 0.001 *****
	Asia	−0.096	0.028	−3.460	**0.001 *****
	Oceania	−0.112	0.032	−3.486	**0.001 *****
	Invasion year	−0.014	0.006	−2.345	**0.019 ***
	Asia × invasion year	0.012	0.011	1.130	**0.259**
	Oceania × invasion year	0.013	0.029	0.448	**0.654**

Topic #	Coefficient	Estimate	Std. error	t value	p value
**7**	(Intercept)	0.039	0.008	4.707	**< 0.001 *****
	Asia	−0.016	0.012	−1.303	**0.193**
	Oceania	−0.020	0.015	−1.358	**0.175**
	Invasion year	−0.004	0.002	−1.509	**0.132**
	Asia × invasion year	0.000	0.005	0.091	**0.927**
	Oceania × invasion year	0.003	0.015	0.221	**0.825**
**8**	(Intercept)	−0.003	0.012	−0.228	**0.820**
	Asia	0.008	0.017	0.458	**0.647**
	Oceania	0.003	0.021	0.136	**0.892**
	Invasion year	0.013	0.004	3.434	**0.001 *****
	Asia × invasion year	−0.008	0.008	−0.992	**0.322**
	Oceania × invasion year	−0.013	0.021	−0.625	**0.532**
**9**	(Intercept)	0.058	0.016	3.726	**< 0.001 *****
	Asia	−0.026	0.021	−1.250	**0.212**
	Oceania	−0.057	0.025	−2.253	**0.025 ***
	Invasion year	−0.011	0.005	−2.292	**0.022 ***
	Asia × invasion year	0.015	0.009	1.614	**0.107**
	Oceania × invasion year	0.011	0.024	0.444	**0.657**
**10**	(Intercept)	0.005	0.010	0.529	**0.597**
	Asia	0.013	0.015	0.864	**0.388**
	Oceania	0.008	0.020	0.400	**0.689**
	Invasion year	0.001	0.003	0.479	**0.632**
	Asia × invasion year	0.004	0.007	0.610	**0.542**
	Oceania × invasion year	0.001	0.024	0.059	**0.953**
**11**	(Intercept)	0.037	0.016	2.356	**0.019 ***
	Asia	0.009	0.024	0.359	**0.720**
	Oceania	−0.034	0.026	−1.324	**0.186**
	Invasion year	−0.003	0.004	−0.584	**0.559**
	Asia × invasion year	−0.013	0.009	−1.414	**0.158**
	Oceania × invasion year	0.002	0.024	0.066	**0.947**
**12**	(Intercept)	0.022	0.015	1.478	**0.140**
	Asia	−0.019	0.022	−0.855	**0.393**
	Oceania	−0.019	0.027	−0.692	**0.489**
	Invasion year	0.007	0.005	1.405	**0.161**
	Asia × invasion year	0.013	0.011	1.124	**0.261**
	Oceania × invasion year	−0.001	0.026	−0.022	**0.983**
**13**	(Intercept)	0.006	0.015	0.413	**0.680**
	Asia	0.035	0.024	1.466	**0.143**

Topic #	Coefficient	Estimate	Std. error	t value	p value
	Oceania	−0.006	0.027	−0.212	**0.833**
	Invasion year	0.009	0.005	1.799	**0.073 .**
	Asia × invasion year	−0.004	0.011	−0.403	**0.687**
	Oceania × invasion year	−0.009	0.027	−0.342	**0.733**
**14**	(Intercept)	0.015	0.012	1.255	**0.210**
	Asia	−0.004	0.018	−0.206	**0.837**
	Oceania	0.009	0.022	0.416	**0.678**
	Invasion year	0.005	0.004	1.467	**0.143**
	Asia × invasion year	−0.006	0.008	−0.793	**0.428**
	Oceania × invasion year	−0.019	0.021	−0.886	**0.376**
**15**	(Intercept)	0.013	0.011	1.114	**0.266**
	Asia	0.011	0.018	0.612	**0.541**
	Oceania	−0.013	0.021	−0.598	**0.550**
	Invasion year	−0.002	0.003	−0.500	**0.617**
	Asia × invasion year	0.015	0.008	1.904	**0.057 .**
	Oceania × invasion year	0.002	0.021	0.079	**0.937**
**16**	(Intercept)	0.015	0.013	1.193	**0.233**
	Asia	0.063	0.021	2.923	**0.004 ****
	Oceania	0.113	0.032	3.513	**< 0.001 *****
	Invasion year	−0.003	0.004	−0.809	**0.419**
	Asia × invasion year	−0.022	0.009	−2.441	**0.015 ***
	Oceania × invasion year	−0.069	0.026	−2.605	**0.009 ****
**17**	(Intercept)	0.011	0.013	0.867	**0.386**
	Asia	0.014	0.020	0.719	**0.472**
	Oceania	−0.010	0.022	−0.441	**0.659**
	Invasion year	0.007	0.004	1.699	**0.090 .**
	Asia × invasion year	−0.007	0.009	−0.827	**0.409**
	Oceania × invasion year	−0.006	0.022	−0.299	**0.765**
**18**	(Intercept)	−0.006	0.011	−0.531	**0.596**
	Asia	0.005	0.016	0.349	**0.727**
	Oceania	0.103	0.029	3.555	**< 0.001 *****
	Invasion year	0.006	0.004	1.660	**0.098 .**
	Asia × invasion year	−0.005	0.007	−0.725	**0.469**
	Oceania × invasion year	0.116	0.045	2.605	**0.009 ****
**19**	(Intercept)	0.017	0.015	1.123	**0.262**
	Asia	−0.010	0.022	−0.462	**0.644**
	Oceania	−0.016	0.026	−0.634	**0.526**
	Invasion year	0.010	0.005	2.031	**0.043 ***

Topic #	Coefficient	Estimate	Std. error	t value	p value
	Asia × invasion year	−0.005	0.010	−0.468	**0.640**
	Oceania × invasion year	−0.011	0.025	−0.421	**0.674**
**20**	(Intercept)	0.003	0.018	0.190	**0.849**
	Asia	0.127	0.031	4.116	**< 0.001 *****
	Oceania	−0.003	0.034	−0.095	**0.925**
	Invasion year	0.003	0.006	0.628	**0.530**
	Asia × invasion year	0.009	0.013	0.694	**0.488**
	Oceania × invasion year	−0.003	0.034	−0.100	**0.921**
**21**	(Intercept)	0.127	0.026	4.908	**< 0.001 *****
	Asia	−0.113	0.032	−3.517	**< 0.001 *****
	Oceania	−0.125	0.038	−3.281	**0.001 ****
	Invasion year	−0.010	0.008	−1.337	**0.182**
	Asia × invasion year	0.011	0.013	0.874	**0.382**
	Oceania × invasion year	0.010	0.033	0.293	**0.769**
**22**	(Intercept)	−0.013	0.015	−0.819	**0.413**
	Asia	−0.009	0.023	−0.409	**0.683**
	Oceania	0.015	0.029	0.505	**0.614**
	Invasion year	0.017	0.005	3.508	**< 0.001 *****
	Asia × invasion year	0.026	0.012	2.147	**0.032 ***
	Oceania × invasion year	0.021	0.039	0.544	**0.586**
**23**	(Intercept)	0.023	0.014	1.677	**0.094 .**
	Asia	−0.014	0.019	−0.706	**0.481**
	Oceania	−0.020	0.025	−0.830	**0.407**
	Invasion year	0.005	0.004	1.129	**0.259**
	Asia × invasion year	−0.001	0.008	−0.130	**0.896**
	Oceania × invasion year	0.034	0.031	1.100	**0.272**
**24**	(Intercept)	0.003	0.015	0.212	**0.832**
	Asia	0.128	0.026	4.913	**< 0.001 *****
	Oceania	0.002	0.029	0.078	**0.937**
	Invasion year	−0.001	0.005	−0.117	**0.907**
	Asia × invasion year	−0.018	0.012	−1.537	**0.125**
	Oceania × invasion year	−0.002	0.029	−0.066	**0.947**
**25**	(Intercept)	0.028	0.012	2.406	**0.017 ***
	Asia	−0.023	0.016	−1.424	**0.155**
	Oceania	−0.009	0.022	−0.418	**0.676**
	Invasion year	−0.004	0.003	−1.090	**0.276**
	Asia × invasion year	0.008	0.007	1.216	**0.224**
	Oceania × invasion year	−0.008	0.019	−0.397	**0.692**

Topic #	Coefficient	Estimate	Std. error	t value	p value
**26**	(Intercept)	0.029	0.015	2.021	**0.044 ***
	Asia	0.081	0.025	3.299	**0.001 ****
	Oceania	−0.027	0.025	−1.075	**0.283**
	Invasion year	−0.005	0.004	−1.323	**0.186**
	Asia × invasion year	−0.031	0.010	−3.102	**0.002 ****
	Oceania × invasion year	0.005	0.025	0.184	**0.854**
**27**	(Intercept)	0.005	0.010	0.474	**0.636**
	Asia	0.011	0.017	0.657	**0.512**
	Oceania	0.070	0.028	2.531	**0.012 ***
	Invasion year	−0.001	0.003	−0.285	**0.776**
	Asia × invasion year	0.001	0.007	0.125	**0.901**
	Oceania × invasion year	0.070	0.035	1.975	**0.049 ***
**28**	(Intercept)	0.009	0.011	0.811	**0.418**
	Asia	0.000	0.016	0.008	**0.994**
	Oceania	0.460	0.038	12.197	**< 0.001 *****
	Invasion year	−0.001	0.003	−0.236	**0.814**
	Asia × invasion year	0.000	0.007	−0.067	**0.947**
	Oceania × invasion year	−0.228	0.036	−6.375	**< 0.001 *****
**29**	(Intercept)	−0.004	0.015	−0.240	**0.810**
	Asia	0.012	0.022	0.528	**0.598**
	Oceania	0.002	0.028	0.086	**0.932**
	Invasion year	0.018	0.005	3.751	**< 0.001 *****
	Asia × invasion year	−0.012	0.010	−1.164	**0.245**
	Oceania × invasion year	0.054	0.039	1.405	**0.161**
**30**	(Intercept)	0.133	0.020	6.609	**< 0.001 *****
	Asia	−0.110	0.027	−4.126	**< 0.001 *****
	Oceania	−0.132	0.031	−4.277	**< 0.001 *****
	Invasion year	−0.021	0.005	−3.790	**< 0.001 *****
	Asia × invasion year	0.019	0.011	1.792	**0.074 .**
	Oceania × invasion year	0.049	0.034	1.454	**0.147**
**31**	(Intercept)	0.005	0.009	0.587	**0.557**
	Asia	−0.015	0.012	−1.216	**0.225**
	Oceania	−0.004	0.016	−0.284	**0.777**
	Invasion year	0.003	0.003	0.852	**0.394**
	Asia × invasion year	0.013	0.006	2.261	**0.024 ***
	Oceania × invasion year	−0.002	0.016	−0.109	**0.913**
**32**	(Intercept)	0.017	0.012	1.399	**0.162**
	Asia	0.013	0.020	0.644	**0.520**

Topic #	Coefficient	Estimate	Std. error	t value	p value
	Oceania	0.012	0.025	0.484	**0.629**
	Invasion year	0.002	0.004	0.596	**0.551**
	Asia × invasion year	−0.004	0.008	−0.478	**0.633**
	Oceania × invasion year	−0.016	0.023	−0.708	**0.479**
**33**	(Intercept)	0.147	0.021	6.891	**< 0.001 *** **
	Asia	−0.069	0.028	−2.473	**0.014 ***
	Oceania	−0.129	0.029	−4.460	**< 0.001 *** **
	Invasion year	−0.024	0.006	−4.366	**< 0.001 *** **
	Asia × invasion year	0.005	0.010	0.489	**0.625**
	Oceania × invasion year	0.021	0.025	0.823	**0.411**

*Note: Africa is the reference category. Significance codes: *** $p < 0.001$, ** $p < 0.01$, * $p < 0.05$, . $p < 0.1$*
